# 

**Published:** 1873-12

**Authors:** 


### Contents of December Number.

### ORIGINAL DEPARTMENT.

ART. I.—Case of Splenic Abscess. By J. Knowles, M. D. .............. 161
ART. II.—Gun-Shot Wound of the Brain. By P. W. Van Peyma, M. D. 164
ART. III.—Synopsis of Essay on Classification of Skin Diseases. By J. W. Southworth, M. D. ............................... 166
ART. IV.—Medical Society of the County of Albany. Annual Meeting. Reported by F. C. Curtis, M. D., Secretary........................ 171

### CORRESPONDENCE.

Fractures of the Cervix Femoris ........................................ 180

### MISCELLANEOUS.

A New Hypodermic Syringe.......................................... 182
A Case of Ovariotomy.............................................. 183
Medical Electricity.................................................... 186
Case of Opium Poisoning. Use of Atropia..................................... 187
Nitrate of Amyl in Epilepsy.......................................... 190
Intra-Recto-Abdominal Exploration................................ 192

### EDITORIAL DEPARTMENT.

Rank of the Medical Corps of the U. S. Army..............................195
Chloroform and Ether..................................................198

BOOKS REVIEWED:

Lectures on Diseases and Injuries of the Ear. By W. B. Dalby, F. R. C. S.......................................... 199
A Practical Treatise on Diseases of the Ear. By D. B. St. John Roosa, M. A., M. D........................... 199
On the Mechanical Treatment of Diseases of the Hip-Joint. By Charles Fayette Taylor, M. D.......................... 200
Books and Pamphlets Received........................................ 200

